# Role of C-reactive protein as a biomarker for prediction of the severity of pulmonary exacerbations in patients with cystic fibrosis

**DOI:** 10.1186/1471-2466-14-150

**Published:** 2014-09-23

**Authors:** Rosa Maria Girón-Moreno, José L Justicia, Sara Yamamoto, Claudia Valenzuela, Carolina Cisneros, Rosa Mar Gómez-Punter, Gilda Fernandes-Vasconcelos, Julio Ancochea

**Affiliations:** Pulmonology Department, la Princesa Institute for Health Research (IP). Hospital Universitario de la Princesa, Madrid, Spain; Medical Affairs Department, Gilead Sciences, Madrid, Spain; Pulmonology Department, Hospital Universitario de la Princesa, Diego de León, n° 62, Madrid, 28006 Spain

**Keywords:** CRP, Cystic fibrosis, Respiratory exacerbation, Hospital admission, *Pseudomonas aeruginosa*, Corticosteroids, Allergic bronchopulmonary aspergillosis, Severity index

## Abstract

**Background:**

Pulmonary exacerbation is one of the main risk factors for death in patients with cystic fibrosis. Several biomarkers have proven useful in the diagnosis and treatment of pulmonary exacerbations, although none has been associated with severity. The objective of the present study was to investigate whether C-reactive protein (CRP) level was associated with the severity of pulmonary exacerbation requiring admission to hospital in patients with cystic fibrosis.

**Methods:**

We designed a severity index for exacerbations based on 4 clinical parameters and determined whether there was an association between CRP levels and severity of the exacerbation. We also investigated the association between CRP and baseline functional and clinical variables.

**Results:**

Twenty-seven patients with cystic fibrosis required 62 admissions to hospital. CRP levels were not significantly associated with the severity index, although they were associated with specific patient characteristics: colonization by *Pseudomonas aeruginosa,* allergic bronchopulmonary aspergillosis, treatment with oral corticosteroids, and number of severe exacerbations treated with intravenous antibiotics during the previous year.

**Conclusions:**

CRP level is not associated with the severity of pulmonary exacerbations, but it is associated with specific clinical characteristics. This simple scoring system (severity index) could prove very useful for evaluating the severity of exacerbations.

## Background

Cystic fibrosis (CF) is the most common fatal autosomal recessive disease in Caucasian individuals. It affects approximately 1 in every 4500 live-born infants, follows a chronic course, and involves several body systems. The main cause of morbidity and mortality continues to be progressive respiratory impairment, which is responsible for 95% of deaths [1]. Respiratory impairment arises from the inability of the mucociliary system to eliminate thick, dehydrated secretions, thus generating bronchial obstruction, inflammation, and recurrent infections that damage the bronchial wall and give rise to bronchiectasis. Patients affected by this chronic disease often present with acute superinfections or exacerbations caused by the emergence of new pathogens, an increase in habitual bacterial load, or the host inflammatory response, each of which leads to impaired respiratory function and a poor prognosis [2–4].

No consensus has been reached on the definition of a pulmonary exacerbation or on diagnostic variables [5–14]. In the absence of objective markers, most published trials define a moderate-severe exacerbation as one requiring admission to hospital or intravenous antibiotics. No validated scoring systems have been designed for early detection of a pulmonary exacerbation, although the clinical picture, which is accompanied by a decrease in lung function parameters, is usually sufficient. Appropriate monitoring of these parameters in a specialized unit and follow-up are key elements for early diagnosis and initiation of suitable immediate therapy [15].

A recent exhaustive review of the usefulness of blood biomarkers of pulmonary exacerbations in patients with cystic fibrosis shows that C-reactive protein (CRP) is the most widely studied marker and that others (neutrophil elastase, antiproteinase complex, interleukin 6, myeloperoxidase, lactoferrin, and calprotectin) seem promising [16].

CRP has high diagnostic sensitivity for inflammatory processes, although its specificity is low. It can prove useful when selecting candidates for treatment or investigating whether or not treatment is efficacious once started. The availability of quantitative methods is important when determining CRP levels.

Several studies have analyzed the value of CRP in the diagnosis of pulmonary exacerbation in patients with cystic fibrosis and in monitoring the response of the exacerbation to treatment [17–31]. The results show that CRP increases during exacerbations and decreases with treatment. However, data on whether CRP can predict or is correlated with severity of pulmonary exacerbations are lacking. Therefore, the primary objective of the present study was to verify whether elevated CRP values at the onset of a pulmonary exacerbation are associated with greater severity of the exacerbation. The secondary objective was to ascertain whether baseline characteristics are associated with CRP values.

## Methods

Our study population comprised all consecutive patients diagnosed with cystic fibrosis who were admitted to La Princesa University Hospital, Madrid, Spain with a moderate to severe pulmonary exacerbation. The study period lasted 3 years (January 1, 2009 until December 31, 2011). This research was approved by the La Princesa Ethics Committee (Madrid, Spain). The patients who signed an informed consent prior to being involved in the study were enrolled. In the case of one of the patients involved who was under eighteen years old this informed consent was signed by one of their parents.

Based on the criteria of Fuchs et al. [5], patients were considered to have had a pulmonary exacerbation if they had at least 4 of the following symptoms or signs: increased cough and expectoration, purulent sputum, hemoptysis, loss of appetite and weight, dyspnea, fever, decrease of >10% in forced expiratory volume in 1 second (FEV_1_) and/or oxygen saturation with respect to baseline values, changes in lung sounds, and radiographic evidence of new infiltrates.

Transplant recipients and patients with associated diseases that could increase CRP levels were excluded (e.g., vasculitis, arthritis, connective tissue disorders, pancreatitis, neoplasm, or heart problems).

We recorded the following clinical variables: sex, age at the first episode, presence of pancreatic insufficiency (defined as fecal elastase levels <200 μg/g and need for pancreatic enzymes), genetic mutations, CF-related diabetes (defined as the need for insulin treatment to control blood sugar levels), allergic bronchopulmonary aspergillosis (if the consensus diagnostic criteria were met [32]), bacterial colonization (isolation of the same germ in 3 consecutive sputum samples separated by an interval of 1 month), continuous treatment received during the 3 months before the episode (macrolides, inhaled corticosteroids, oral corticosteroids, ibuprofen, nebulized antibiotics, DNase, or hypertonic saline solution), and the number of exacerbations during the previous year (specifying whether antibiotic therapy was oral or intravenous).

Lung function values were collected at baseline and at the end of intravenous antibiotic therapy. Baseline lung function was defined as the best value from the previous year. All patients underwent spirometry at the clinic (Datospir 120, Síbel); FEV_1_ and forced vital capacity (FVC) were expressed as absolute values (liters) and as a percentage of the predicted value. The FEV_1_/FVC ratio was calculated.

A chest radiograph was taken at admission and compared with the previous radiograph to detect new pathologic findings. We also recorded the intravenous antibiotics taken to treat the exacerbation and the number of days on intravenous therapy, specifying the number of days with treatment at hospital and at home (hospitalized patients whose symptoms improved were discharged and continued treatment at home). Complications during the exacerbation were recorded (i.e., hemoptysis, pneumothorax, respiratory insufficiency, need for noninvasive mechanical ventilation, need for invasive mechanical ventilation, and death).

A blood sample was taken during the first 48 hours to obtain the following parameters: total leukocytes, erythrocyte sedimentation rate (ESR), immunoglobulins (IgG, IgM, and IgA), fibrinogen, and CRP. This last one was measured by immunoturbidimetric assay (Tina-quant CRP detection method; Roche Diagnostics) at our hospital clinical laboratory.

### Pulmonary exacerbation severity index

An index was constructed to determine the severity of pulmonary exacerbations. The index was based on 4 parameters:

- Loss of lung function after an exacerbation:

(baseline FEV_1_ – initial FEV_1_)/baseline FEV_1_, scored as follows: <15% = 0; ≥15% = 1

b. Recovery of lung function after an exacerbation:

Final FEV_1_/baseline FEV_1_, scored as follows: ≥90% = 0, < 90% = 1

c. Number of days receiving intravenous antibiotics in hospital

≤ 14 days = 0, > 14 days = 1

d. Complications:

No = 0, Yes = 1

Baseline FEV_1_ corresponded to the value of FEV_1_ during the stable phase (the best value during the previous year), initial FEV_1_ to the FEV_1_ value at the initiation of antibiotic therapy administered to treat the exacerbation, and final FEV_1_ to the FEV_1_ value at the end of antibiotic therapy. Number of days receiving IV antibiotics in hospital was chosen as one of the items instead of total number of days of IV antibiotics (including antibiotics at home) because the first one reflects more appropriately the severity of an exacerbation: many times in clinical practice, patients are discharged from hospital but they keep routinely taking medicines for some days in spite of the fact that their exacerbation is considered resolved.

The values of the index ranged from 0 to 4. Patients with values of 3 and 4 were grouped together owing to the small number of patients in each of the original groups; therefore, the possible values for the index were 0, 1, 2, and 3.

### Statistical analysis

The results were analyzed using SPSS version 19 (SPSS Inc). Normality was verified using the Kolmogorov-Smirnov test; in the case of a non-normal distribution, nonparametric tests were used (Spearman correlation, Mann–Whitney, and Kruskal-Wallis). In the descriptive analysis, the quantitative variables were expressed as mean (SD) and the qualitative variables as percentages. When a patient suffered multiple pulmonary exacerbations along the study period, each exacerbation was analyzed as a separate event. The percentage of loss and recovery of FEV_1_ was calculated using the formulas described above. Initial CRP values were associated with the severity index and with the following baseline clinical variables: sex, age, genotype, bacterial colonization, pancreatic insufficiency, diabetes, allergic bronchopulmonary aspergillosis, previous treatment, number of exacerbations, and baseline FEV_1_. A sensitivity analysis of the association between CRP and the severity index was performed selecting the exacerbations with CRP measurements within 24 hours after hospital admission. Multivariable analysis using linear regression (stepwise variable selection method) was conducted with baseline clinical variables showing statistically significant association with CRP.

Statistical significance was set at p < 0.05.

## Results

The study population comprised 27 patients with cystic fibrosis aged 15 to 45 years, each of whom had experienced at least 1 pulmonary exacerbation during the study period. The colonizing microorganism was *Pseudomonas aeruginosa* in 85.2% of cases; mean baseline FEV_1_ (% predicted) was 53.2% (Table 1).

**Table 1:** **Clinical characteristics of the patients (n = 27)**

Clinical variable	Value
Age, y mean (SD)	25.9 (6.7)
Sex, man/woman (n)	10/17
Lung function tests: mean (SD)	
FVC, L	3.0 (0.84)
FVC, % predicted	73.8 (18.8)
FEV_1_, L	1.9 (0.6)
FEV_1_, % predicted	53.2 (16.3)
FEV_1_/FVC	61.1 (11.3)
Genetic mutation, n (%)	
F508del/F508del	8 (29.6)
F508del/other	15 (55.6)
Other	4 (14.8)
Diabetes, n (%)	5 (18.5)
ABPA, n (%)	11 (40.7)
Pancreatic insufficiency, n (%)	24 (88.9)
Bacterial colonization, n (%)	
*Staphylococcus aureus*	18 (66.7)
*MRSA*	4 (14.8)
*Pseudomonas aeruginosa*	23 (85.2)
*Burkholderia cepacia*	3 (7.4)
*Achromobacter xylosoxidans*	6 (21.7)
*Haemophilus influenzae*	1 (3.7)
*Stenotrophomonas maltophilia*	1 (3.7)
Treatment, n (%)	
Macrolides	22 (81.5)
Inhaled corticosteroids	21 (77.8)
Oral corticosteroids	5 (18.5)
Nebulized antibiotics	23 (85.2)
DNase	14 (51.9)
Hypertonic saline solution	13 (48.1)
No. of exacerbations during the previous year treated with oral antibiotics, mean (SD)	3.2 (1.9)
No. of exacerbations during the previous year treated with intravenous antibiotics, mean (SD)	1.77 (1.73)

FVC, forced vital capacity; FEV_1_, forced expiratory volume in the first second; ABPA, allergic bronchopulmonary aspergillosis; MRSA, methicillin-resistant *Staphylococcus aureus*.

**Figure 1:**
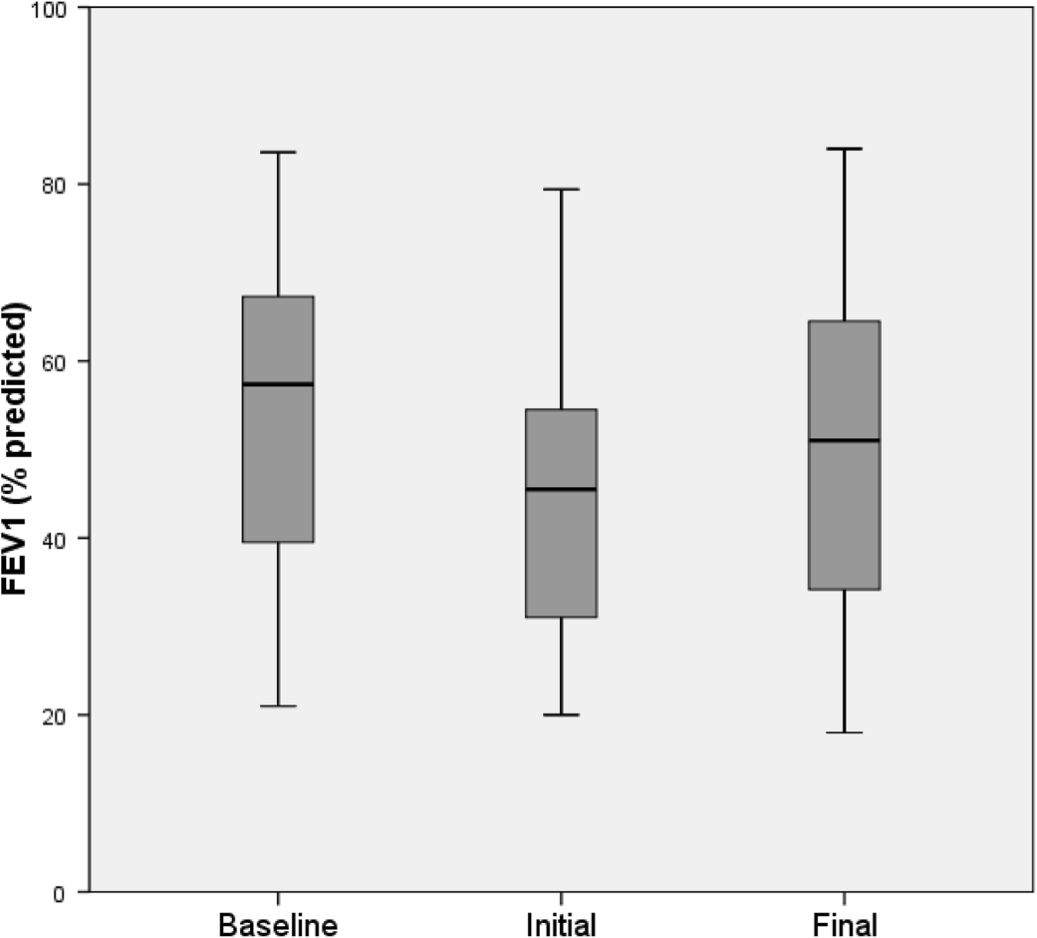
**FEV**_**1**_ **(% predicted) before and after exacerbations.** The boxes represent the middle 50% of patients; the whiskers include all patients in each group. The horizontal line within the box represents the median FEV_1_. The x axis represents the best FEV_1_ in the 12 months before admission (baseline); FEV_1_ at hospitalization (initial); and the best FEV_1_ at the end of antibiotic therapy (final).

**Figure 2:**
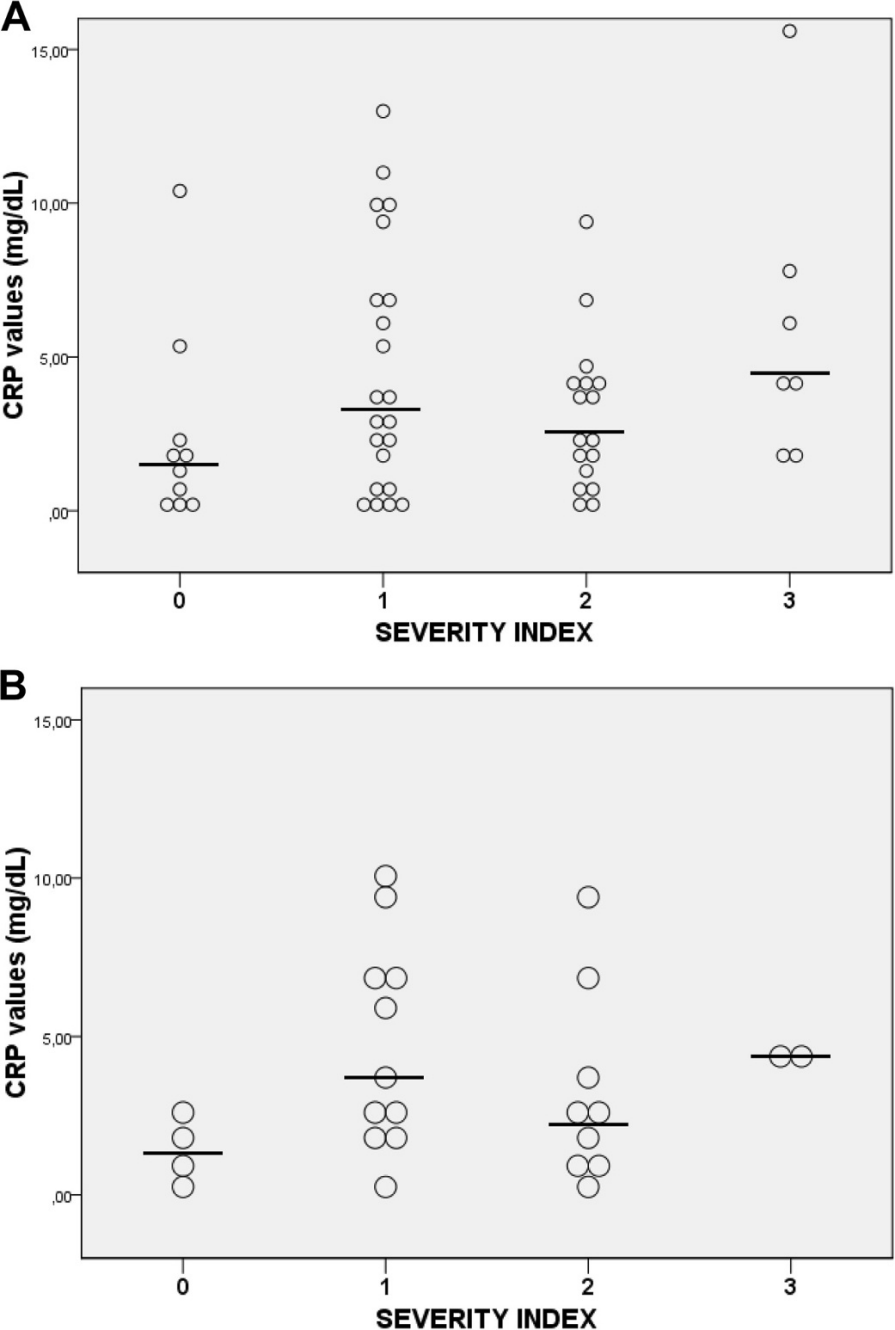
**Correlation between C-reactive protein and pulmonary exacerbation severity index. A**. Correlation between CRP and severity index for all the exacerbations. **B**. Correlation between CRP and severity index for exacerbations with CRP measurement within 24 hours after hospital admission. The circles represent the values of CRP at the beginning of each exacerbation. The x axis represents the group of exacerbations according its severity index. The horizontal line within the group represents the median CRP value.

During the study period, the 27 patients experienced 62 exacerbations requiring admission to hospital and intravenous antibiotic treatment (tobramycin in 61% of cases, ceftazidime in 37%, meropenem in 32%, and imipenem in 30%). Prior to admission, 25 (40.3%) exacerbations were treated with oral antibiotics (19.4% with quinolones, 9.7% with amoxicilin, 8.1% with co-trimoxazole and 3.2% with others) for 3 to 7 days in 9 (14.5%) cases, 8 to14 days in 11 (17.7%), and 21 days in 5 (8.1%). On average, the fall in FEV_1_ after the exacerbation was approximately 10% and the subsequent recovery only 7% (Figure 1). Some markers of inflammation were increased (CRP, ESR, and fibrinogen). Patients were hospitalized for a mean of 10.4 days and received intravenous antibiotic therapy for 17 days (Table 2).CRP was measured within the first 24 hours after hospital admission in half of the exacerbation episodes; in the other half CRP was obtained at 48 hours. The correlation between CRP and the severity index was not statistically significant in either the whole sample (Spearman’s rho, 0.203; p = 0.134) or the subgroup with CRP measurements within 24 hours (Spearman’s rho, 0.193; p = 0.344) (Figure 2). No correlation between the severity index and other inflammatory markers (leukocytes, ESR, IgG, IgA, IgM and fibrinogen) was found (data not shown).

**Table 2:** **Characteristics of the exacerbations (n = 62)**

Variable	Value
Lung function tests, mean (SD)	
Initial FEV_1_, L	1.50 (0.5)
Initial FEV_1_, % predicted	43.9 (15.6)
Final FEV_1_, L	1.7 (0.5)
Final FEV_1_, % predicted	50.3 (17.3)
C-reactive protein, mg/dL, mean (SD)	
Severity index, 0 (n = 10)	2.4 (3.2)
Severity index, 1 (n = 22)	4.5 (4.0)
Severity index, 2 (n = 17)	3.0 (2.4)
Severity index, 3 (n = 7)	5.9 (4.8)
Other markers of inflammation, mean (SD)	
Leukocytes, cells/mL	10,532 (4,738)
ESR, mm/h	45.8 (24.2)
Total non-specific IgG, mg/dL	1371.8 (408.7)
Total non-specific IgA, mg/dL	298.6 (94.1)
Total non-specific IgM, mg/dL	155.9 (80.9)
Fibrinogen, mg/dL	547.1 (135.3)
Hospital stay, days, mean (SD)	10.4 (6.5)
Home intravenous treatment, days, mean (SD)	6.7 (4.9)
Chest radiograph, n (%)	
Not done	1 (1.6)
Worse	25 (40.3)
Similar to previous	36 (58.1)
Main complications during exacerbations, n (%)	
Hemoptysis	13 (21)
Pneumothorax	2 (3.2)
Respiratory insufficiency	24 (38.7)
Noninvasive mechanical ventilation	1 (1.6)
Invasive mechanical ventilation	1 (1.6)

FEV_1_, forced expiratory volume in the first second; ESR, erythrocyte sedimentation rate; Ig, immunoglobulin.

Mean values of CRP were similar in patients previously treated or not treated with oral antibiotics (4.2 vs 3.8; p = 0.405). Duration of previous treatment with oral antibiotics didn’t seem to influence the CRP values either: 4.8 mg/dL, 3.6 mg/dL, and 4.4 mg/dL in patients treated for 3 to7 days, 8 to 14 days, and 21 days, respectively (p = 0.823).

Analysis of the association between CRP and baseline characteristics revealed higher CRP levels in patients colonized by *P. aeruginosa* (4.4 vs 2.2; p = 0.016), patients diagnosed with allergic bronchopulmonary aspergillosis (4.9 vs 2.9; p = 0.024), patients taking oral corticosteroids (6.1 vs 3.0; p = 0.010), and patients who had had a larger number of exacerbations treated with intravenous antibiotics during the previous year (p = 0.001). Association between CRP and the rest of baseline characteristics was not statistically significant. After the multivariable analysis, only treatment with oral corticosteroids was identified as independent factor associated with CRP values (beta coefficient = 3.013; p = 0.005).

## Discussion

Pulmonary exacerbations in patients with cystic fibrosis can lead to a gradual decline in lung function, impaired quality of life, and increased health care costs. In addition, they have a negative impact on prognosis, since survival decreases [1–4]. In the present study, we investigated the potential association between CRP level and the severity of pulmonary exacerbations requiring hospital admission in patients with cystic fibrosis and ascertained whether CRP level was associated with specific clinical characteristics.

In the absence of validated scoring systems, we attempted to meet our first objective by determining the severity of exacerbations through the design of a simple, user-friendly score based on 4 parameters considered important when evaluating the severity of an exacerbation in clinical practice. We used these parameters to construct a severity index with values ranging from 0 to 4, in which the least severe exacerbation was scored 0 and the most severe 4. In contrast with the proposed hypothesis, CRP levels were not significantly associated with the severity index.

A recent and exhaustive systematic review of all studies in which blood biomarkers of respiratory exacerbations in CF patients were analyzed concluded that CRP was the most widely studied marker [16]. CRP levels usually increase significantly at the onset of an exacerbation [17–21], although this parameter does not enable us to identify the causative microorganism [19, 20, 22, 23]. The role of CRP in predicting the duration, frequency, and recurrence rate of pulmonary exacerbations has also been evaluated [24–26], and an inverse association between CRP and time until the subsequent exacerbation has been reported [26]. The most widely studied aspect of CRP is its role in the response to treatment of an exacerbation. Many studies show a significant decrease in CRP after antibiotic treatment [17, 19, 20, 22–25, 27–31]. However, to date, the usefulness of biomarkers for determining the severity of a pulmonary exacerbation has not been evaluated [16], thus giving more weight to the hypothesis proposed in the present study, namely, that the increase in CRP is greater in more severe exacerbations.

The fact that we did not find a significant association between CRP values and the severity of an exacerbation leads us to think that this biomarker may not be useful for measuring the severity of pulmonary exacerbations in patients with cystic fibrosis or that it is only useful in specific subgroups. For example, Ibrahim et al. [33] analyzed 628 episodes of exacerbation and found that a subgroup of patients with higher FEV_1_ values, less impaired lung function, and no colonization by *P. aeruginosa* had normal CRP levels and needed fewer days of treatment. The authors concluded that very aggressive regimens should not be used in this subgroup. As the plasma half-life of CRP is about 19 hours [34], CRP levels might fall quickly in patients responding to treatment and the CRP value might have less ability to predict exacerbation severity if the measurement was delayed. However, the analysis in the subgroup of exacerbations which CRP values were early obtained (first 24 hours) did not show relationship between CRP and the severity index.

When we analyzed the association between CRP and baseline characteristics, we found a significant association with colonization by *P. aeruginosa*, diagnosis of allergic bronchopulmonary aspergillosis, current treatment with oral corticosteroids, and the number of exacerbations requiring intravenous antibiotic treatment during the previous year.

Our results show that patients colonized by *P. aeruginosa* have higher CRP levels, as reported by Watkin et al. [22], who concluded that patients intermittently colonized by other bacteria, such as *Haemophilus influenzae* or *Staphylococcus aureus*, had lower CRP levels. A recent publication on inflammation and microbiota in pulmonary exacerbations in patients with cystic fibrosis found that those who had a high concentration of *P. aeruginosa* colonies had lower FEV_1_ values and higher CRP values, in contrast with the remaining bacteria studied [35].

In cystic fibrosis, recurrent infection and obstruction of the airways lead to inflammation, lung damage in the long term, respiratory insufficiency, and death [36]. Given that inflammation is present from onset, anti-inflammatory drugs such as corticosteroids could prove useful for delaying impairment of lung function in the long term. However, owing to the severe side effects of these drugs, long-term use is not recommended, except in very specific cases. Paradoxically, in our study, patients who received oral corticosteroids, which have a strong anti-inflammatory effect, had higher CRP levels than patients who did not. In fact, this variable was the only one that remained significant after conducting a multivariable analysis. This observation could be explained by the fact that corticosteroid therapy is likely initiated in patients with greater respiratory impairment and increased levels of inflammatory markers.

Our study is subject to a series of limitations. First, the sample size is small owing to the low prevalence of cystic fibrosis and to the fact that it was performed in a single hospital. In order to definitively rule out the hypothesis established, we should perform a multicenter study with a higher number of patients and pulmonary exacerbations. Second, we did not analyze more complex inflammatory markers or perform serial determinations of CRP, both of which would have generated more robust conclusions. For example, neutrophil elastase and interleukin-8 are important biomarkers in detecting and monitoring pulmonary exacerbations [16, 37, 38] so they may be more relevant to the severity index than CRP. Finally, we used a non-validated scoring system (severity index) and therefore it might not be comprehensive enough to evaluate the severity of the exacerbations; nevertheless, we consider the score to be very useful, since it enabled us to classify the intensity of the exacerbations.

## Conclusion

Our findings enable us to conclude that CRP levels are not associated with the pulmonary exacerbation severity index but that they are associated with some clinical variables, such as colonization by *P. aeruginosa*, number of exacerbations treated with intravenous antibiotics during the previous year, treatment with oral corticosteroids, and allergic bronchopulmonary aspergillosis. A simple scoring system, such as the severity index, could prove useful for classifying the severity of pulmonary exacerbations in patients who require intravenous treatment and would enable us to act accordingly.

## Ethics

The research has been performed in accordance with the Declaration of Helsinki and been approved by the Ethic Committee of Hospital de la Princesa PI-460. All the patients signed an informed consent to authorize the publication of the data.
